# The burden of prolonged indwelling catheter after acute urinary retention in Ikeja – Lagos, Nigeria

**DOI:** 10.1186/1471-2490-7-16

**Published:** 2007-09-26

**Authors:** Stephen O Ikuerowo, Aderinsola A Ogunade, Taiwo O Ogunlowo, Charles C Uzodimma, Julius O Esho

**Affiliations:** 1 Urology Unit, Department of Surgery, Lagos State University College of Medicine and, Lagos State University Teaching Hospital Ikeja, Lagos, Nigeria; 2 National Orthopedic Hospital, Igbobi, Lagos, Nigeria

## Abstract

**Background:**

Acute urinary retention (AUR) is a common urological problem. We have observed a growing list of patients on indwelling bladder catheter awaiting surgery after AUR. This study was aimed at identifying the health, financial and quality of life (QoL) implications of prolonged use of indwelling catheter in these patients

**Methods:**

We review the side-effects, QoL and cost of changing an indwelling catheter among patients who were on the waiting list for definitive surgery after AUR. All the 62 patients who presented to weekly catheter clinic for change of the indwelling catheter were recruited over a 3 – week period into the study.

**Results:**

The mean age of the patients was 57.5 years and the mean catheter use time was 23 months. The aetiology of AUR was BPH in 40 (64%) and urethral trauma in 16 (28.4%) of the patients. The common side effects of prolonged catheterization included urethral/suprapubic pain, bleeding per urethram, loss of dignity, loss of job or being out of school, lack of sexual intercourse, pericatheter leakage of urine and recurrent urinary tract infection. The cost of change of the indwelling catheter to the patient each time ranged from 460.00 – 2500.00 Naira (averaged 789.67 Naira). The total annual cost for the change of indwelling catheter after AUR in our catheter clinic was estimated to be 7,350,000.00 Naira (58,800 US dollars) with 1,890,000.00 Naira (15,120 US dollars) being the cost borne by the patients per annum and the rest being government subsidy. Fifty-three (85.5%) patients described that they were unhappy. There was a significant correlation between QoL and the presence of pain (p = 0.015) and bleeding (p = 0.042) associated with the presence of an indwelling catheter.

**Conclusion:**

The need to have an indwelling catheter for a prolonged period after AUR is a painful experience and associated with several side-effects. This has a significant negative effect on the patients' QoL and constitutes a significant financial burden to the patients and the government. We suggest that measures should be put in place to reduce the waiting time for surgery and therefore the catheterization time among the patients with AUR.

## Background

Acute urinary retention (AUR) is a common urological emergency characterized by a sudden and painful inability to pass urine. It is estimated that 10% of men in their seventies and a third in their eighties will have AUR within the next five years [1,2]. AUR is an important public health issue. It has a significant impact on the patient's health-related QoL and is associated with a substantial economic burden [1,2].

AUR may be caused by: a greater resistance to the flow of urine due to mechanical or dynamic obstruction, bladder over-distension or neuropathic disease [1]. The immediate treatment of AUR consists of bladder decompression, either by a urethral catheter or a suprapubic catheter [1,2]. In our practice, suprapubic cystostomy is usually reserved for patients in whom urethral catheterization is contraindicated or has failed. The subsequent management depends on the cause of the AUR. The patients often have to remain on a long waiting list for the definitive surgical procedure [3].

The aim of the study was to describe the problems associated with the prolonged use of indwelling catheter as well as the cost implication of changing these catheters among patients who previously had AUR while awaiting definitive surgical treatment in our institution.

## Methods

We evaluated all the patients who presented for a change of their indwelling catheter at the weekly catheter clinic of Lagos State University Teaching Hospital, Ikeja, Lagos, Nigeria, over a 3-week period in the month of May, 2006. Approval for the study was obtained from our institution review board and the patients gave their consent after the study had been explained to them. The patients have been on indwelling bladder catheter following AUR. Our patients usually present to the catheter clinic at a regular interval of 3 weeks for a change of the indwelling catheter. Therefore the study was limited to a 3-week period so as to avoiding re-evaluating patients who had already been seen previously. A questionnaire was designed and completed for all the patients. Initial validation of the questionnaire was carried out on 10 patients before it was applied. The house physician assisted the patients who had difficulty completing the questionnaire. Information obtained included the patients' biodata, clinical diagnoses, duration of catheter use, reason for and effect of prolonged catheter use, estimated cost of change of catheter and the patients' perceived QoL.

The cost of change of catheter was estimated by the patients. This included the cost of procurement of all the materials needed for the catheter change, the cost of drugs prescribed in relation to the catheter change and the cost of transportation to and from the hospital. The patients did not pay any service charge as this is taken care of by the subsidy from the government.

As regards the patients' perceived QoL, the question ''How do you feel about your life regarding the need to use an indwelling bladder catheter while awaiting operation?" was asked. The responses were ''sad", ''indifferent", or ''happy". We did not use the QoL measure in the International Prostate Symptoms Score questionnaire because of the difficulty in interpreting some of the responses in our local languages. Further information was also obtained from the patients' notes.

The data was evaluated using the SPSS 14.0 software for Windows. Comparison of means was carried out using the Student's t-test and correlations of parametric and non-parametric data were done using Pearson's and Spearman's rho tests respectively. *p *≤ 0.05 was considered significant.

## Results

There were 62 patients who presented to the catheter clinic for a change of the indwelling catheter over the 3 – week period of the study. All the patients were males. The mean age of the patients was 57.5 years (range 13 – 85 years). Fifty-two (83.9%) were married and 10 (16.1%) were single before they started using the catheter. Thirty-one patients (50%) had indwelling urethral catheter and the other 31 (50%) used a suprapubic cystostomy catheter. Over this 3-week period, only 4 (6.5%) patients were new clinic attendees. The other 58 (93.5%) patients had attended the catheter clinic for a change of catheter more than once. The mean duration of use of catheter was 23 months (range 0.5 – 72 months). There was no significant difference in the duration of use of catheter between the patients who used urethral catheter (mean 19.2 months) and the patients who used suprapubic catheter (mean 23.5 months) {p = 0.412}. The patients changed their catheters at a mean interval of 2.92 (± 1.08 SD) weeks.

The clinical diagnoses of these patients who attended the catheter clinic are summarized in table 1. Forty (64.5%) patients had a clinical diagnosis of benign prostatic hyperplasia (BPH) as the cause of the AUR and were awaiting prostatectomy. Twenty-nine (72.5%) of these patients with BPH had urethral catheter and 11 (27.5%) had suprapubic cystostomy catheter. Of these patients, only 13 (32.5%) had ever had trial without catheter (TWOC) carried out. There were 6 (46.2%) patients whose TWOC was initially successful and the patients were able to void after removal of the catheter. These six patients however had a recurrence of the AUR within the next six months and had to be re-catheterized. Urethral trauma was the second commonest cause of AUR constituting 16 (25.8%) of the patients. The patients developed urethral stricture subsequently.

**Table 1:** Aetiology of AUR in 62 patients

**Clinical diagnosis**	**N**	**%**	**Mean duration of catheter use (months)**
BPH	40	64.0	20.4
Urethral trauma	16	28.4	23.7
Post-inflammatory stricture	5	6.0	23.2
Prostate cancer	1	1.6	--

The side-effects that were associated with the prolonged use of indwelling catheters are summarized in table 2. Pain along the urethra or suprapubic pain associated with presence of an indwelling catheter was the commonest side-effect reported by the patients and occurred in 43 (69.4%) patients. The pain had a significant negative impact on the patients' health-related QoL due to the presence of an indwelling catheter. Thirty-nine (62.9%) patients complained of loss of dignity associated with the use of catheter. The complaint of loss of dignity did not however significantly influence the overall health-related QoL. Eighteen (29%) patients were either out of work or out of school mainly because of pain from the indwelling catheter (p = 0.004) or because they could not bear the shame of wearing an indwelling catheter (p = 0.025). Bleeding was a common side-effect among these patients on prolonged use of indwelling catheter occurring in 20 (32.3%) patients. Inability to have sexual intercourse was another major side-effect reported by the patients. Twenty-three (37.1%) patients reported that lack of sexual intercourse was a serious bother to them. This occurred equally among the patients who used urethral or suprapubic catheter (p = 0.496). Seven of these patients have been deserted by their wives or sexual partners because of the inability to perform satisfactory sexual intercourse. Pericatheter leakage of urine was reported by 10 (16.1%) patients.

**Table 2:** Problems associated with prolonged catheter use in all 62 patients

**Problems**	**Frequency (n/%)**	**Correlation with QoL (*p-value*)**
Pain	43 (69.4)	0.015, 0.049*
Bleeding	20 (32.3)	0.170, 0.042*
Loss of dignity	23 (37.1)	0.805
No sexual intercourse	23 (37.1)	0.451
Out of work or school	18 (29.0)	0.764
Pericatheter leakage of urine	10 (16.1)	0.567
Recurrent symptomatic UTI	3 (4.8)	0.474

QoL = Quality of life, UTI = urinary tract infection, *BPH patients only

The average cost of change of catheter was estimated by the patients. It must be noted that public health services in Lagos State is highly subsidized. The mean cost of change of indwelling catheter each time for the patient was 789.67 Naira (range 460 – 2500 Naira). The service charge paid in non-government hospitals in the same environment for a change of indwelling bladder catheter averages 5,000.00 Naira. This is however covered by the government subsidy on health care among patients seen in our hospital. Therefore, for a change of catheter in each patient, it would cost between 5,460.67 Naira to 7500.00 Naira if they were to pay the service charge as well. The total cost for the change of catheter for all 62 patients over 3 weeks was 109,032.00 Naira (or 424,038.00 Naira when the service charge would be added). Extrapolating the cost for 1 year, this would amounts to 1,890,000.00 Naira as cost borne by the patients and 5,460,000.00 Naira as the service charge covered by the subsidy from the government. A total of 7,350,000.00 Naira would therefore be the cost per year to change indwelling bladder catheter among patients who had acute urinary retention in our hospital while awaiting definitive surgery. This is equivalent to 58,800 US dollars per annum.

The patients were asked to describe how they feel about their life regarding the need to use a catheter for a prolonged period while awaiting surgery as ''sad", ''indifferent" or ''happy". Fifty-three (85.5%) patients described that they were sad, 6 (9.7%) were indifferent and 3 (4.8%) patients were happy. Overall, there was a significant correlation between the patient's perceived health-related QoL and the presence of pain in the suprapubic area or along the urethra associated with the indwelling catheter (p = 0.015 Spearman's rho test). Furthermore, when only patients with BPH were considered, pain (p = 0.049) and bleeding (p = 0.042) had a significant negative impact on the health-related QoL.

## Discussion and conclusion

AUR, a common urological emergency, is for the patient a dreaded consequence of the progression of lower urinary tract symptoms (LUTS). AUR contributes significantly to the QoL of the patients with LUTS [1]. It is usually managed by immediate urethral or suprapubic catheterization. Patients who fail a trial without catheter (TWOC) following AUR are usually planned to have definitive surgical treatment [2].

In the more developed parts of the world, patients who fail TWOC after AUR have an average catheterization time of 12 days before the definitive surgical procedures are carried out [4]. This is a far cry from the result of this study that reveals an average catheterization time of 23 months and majority of these patients would still wait for more time before having surgery. A study in Ibadan, Nigeria also shows a similar prolonged waiting time before surgery in patients with BPH [3]. The duration of catheterization in the study ranged from 1 to 13 months with a mean of 8 months. Catheter-associated complications occurred in 37.5% of patients in this particular study in Ibadan [3]. This underscores not only the need for more operation time for urologic procedure to be made available in our public hospitals but also the need to have more urologists to serve the teaming population of urological patients in the public hospitals of Lagos, Nigeria. It is common knowledge that patients who have the financial power to afford the treatment in the non-government hospitals in Lagos would not need to have these prolonged catheterization and waiting time. Therefore, it is possible that when the National Health Insurance Policy becomes fully operational, more patients may be able to afford treatment in the non-government hospital. This may then lead to a lot of the pressures being taken off the public hospital by patients who may not ordinarily be able to afford treatment in the non-government hospital setting.

Prolonged use of indwelling catheter is accompanied by several side-effects and complications [4, 5] as also demonstrated by this study. Complications or side-effects occurred in 95% of patients following prolonged use of indwelling catheter after AUR. Most patients would have urethral and/or suprapubic pain, occurring in 69% of patients. Bleeding is also a common complication especially in patients with benign prostatic hyperplasia. In addition to the fact that most patients (85.5%) described that they are sad with their life, we also found that the reason for the sadness is often because of pain and bleeding associated with the prolonged use of indwelling catheter. Furthermore, the cost implication of having an indwelling bladder catheter is enormous as revealed in this study. In addition to the huge subsidy that is given by the state government, the need to regularly change the indwelling catheter is a huge financial burden to majority of these patients. It is clearly evident that a considerable savings in resources could be achieved if catheterization time after AUR is reduced.

In conclusion, AUR is not only a painful experience for the patient, the need to wear an indwelling catheter for a prolonged period while awaiting surgery is also accompanied with pain in majority of the patients. The prolonged use of an indwelling catheter in addition to its huge financial burden also has several other complications and significantly contributes to patients' poor QoL. Measures that would significantly reduce the catheterization time and consequently the waiting time for surgery will therefore be highly desirable.

## Competing interests

The author(s) declare that they have no competing interests.

## Authors' contributions

ISO made substantial contributions to conception and design, analysis and interpretation of data; drafting the manuscript, revising it critically for important intellectual content. OAA, OTO, UCC and EJO made substantial contributions to design, data collection, and revision of the manuscript. All the authors read and gave approval for the final version of the manuscript.

## Pre-publication history

The pre-publication history for this paper can be accessed here:


